# Sociodemographic Factors Associated with Adolescents’ and Young Adults’ Susceptibility, Use, and Intended Future Use of Different E-Cigarette Devices

**DOI:** 10.3390/ijerph19041941

**Published:** 2022-02-09

**Authors:** Shivani M. Gaiha, Poonam Rao, Bonnie Halpern-Felsher

**Affiliations:** 1 Stanford Reach Lab, Division of Adolescent Medicine, Department of Pediatrics, Stanford University, Stanford, CA 94304, USA; gaiha@stanford.edu; 2 Center for Tobacco Control Research and Education, University of California San Francisco, San Francisco, CA 94117, USA; Poonam.Rao@ucsf.edu

**Keywords:** e-cigarette, adolescent, young adult, sociodemographics, LGBTQ, race/ethnicity, device, disposable, pod/cartridge-based, mods

## Abstract

Numerous studies have identified sociodemographic factors associated with susceptibility, ever-use and past-30-day use of e-cigarettes, including JUUL. However, it remains unknown which sociodemographic factors are associated with adolescents’ and young adults’ (AYA) use of the entire spectrum of different types of e-cigarette devices (e.g., disposables, pod/cartridge-based, and other e-cigarettes, like mods or tanks). The aim of this study was to examine the relationship between sociodemographic factors and use, future use intent and susceptibility to use different e-cigarette device types. We conducted a national online survey using a convenience sample of 13–24-year-olds, 50:50 e-cigarette ever- to never-users and sex and race/ethnicity balanced per the U.S. Census (n = 4351). Sociodemographic factors were not associated with ever use of disposables among AYAs or generally with intent to use e-cigarette devices in the future. However, sociodemographic factors were related to the use of pod/cartridge-based and other e-cigarette devices. LGBTQ+ AYAs were more likely to use pod/cartridge-based devices and to be susceptible to using all device types compared to other AYAs. Young adults, males, and other/multiracial non-Hispanic AYAs were more likely to report past-30-day-use of all devices and AA/Black non-Hispanic AYAs were more likely to report past-30-day use of pod/cartridge-based and other devices compared to former users. AA/Black non-Hispanic AYAs were more likely to be susceptible to using all devices and other/multiracial non-Hispanic AYAs were susceptible to using other devices (compared to White non-Hispanic AYAs). AYAs under 21 who were former users were more likely to intend using other devices in the future compared to AYAs 21 years or above. These findings may inform targeted prevention efforts to curb the growing popularity of different devices among AYAs.

## 1. Introduction 

Electronic cigarettes (e-cigarettes) first came on the U.S. market in 2007, and since then we have seen a proliferation of different types of e-cigarette device types, including cig-a-likes, pens, mods, tanks, pod/cartridge-based devices made popular by JUUL, and disposables [1,2,3]. Recent data suggest that in the past 30 days, adolescents and young adults (AYAs) most commonly used disposable devices, such as Puff Bar, while pod/cartridge-based e-cigarettes also remained popular [3,4,5]. E-cigarettes generally contain chemicals and constituents, including nicotine, solvents and flavors, that are associated with harms to the heart, lungs, and brain, as well as mental health [4,6,7,8,9,10,11,12,13,14,15]. Nevertheless, in 2021, over 2 million U.S. middle and high school students reported using e-cigarettes in the past 30 days [4]. One in four young adults (24.5%) reported using e-cigarettes as of 2019 [16]. Although studies have examined e-cigarette device types used by adolescents and young adults (AYAs) [4,5], we know little about the sociodemographic factors associated with use of different e-cigarette device types.

Overall, studies examining sociodemographic factors associated with e-cigarette use have shown that younger AYAs are more likely to be susceptible to using e-cigarettes [17] and JUUL [18] and are more likely to have ever used JUUL in the past 30 days [18,19]. In addition, albeit with varying definitions, sexual minority adolescents compared to heterosexual adolescents or AYAs identifying as LGBTQ+ compared to non-LGBTQ+ were more likely to be susceptible to using e-cigarettes [18], and were more likely to have ever used [20,21] and used e-cigarettes in the past 30 days [21]. Other studies show that Hispanic compared to non-Hispanic White AYAs were more likely to be susceptible to using e-cigarettes [17], and that ever using JUUL and using JUUL in the past 30 days was less likely among Black/African American non-Hispanic AYAs compared to non-Hispanic White AYAs [19]. Further, although AYAs identifying as female compared to male were more likely to be susceptible to using e-cigarettes [17], a study showed that males were more likely to have used JUUL in the past 30 days compared to females [18]. Finally, those who just met basic expenses were less likely to use e-cigarettes compared to those who lived comfortably [19]. Notably, one study showed sociodemographic factors associated with JUUL and other e-cigarette devices, but did not separate or specify the “other e-cigarette devices” as disposables, other (non-JUUL) brands of pod/cartridge-based devices or other e-cigarette devices, like mods [18].

While studies have examined sociodemographic factors associated with susceptibility to use e-cigarettes among never-users and among ever and past-30-day users of e-cigarettes and JUUL, studies have not assessed sociodemographic factors across the entire spectrum of AYA e-cigarette use, including susceptibility, initiation, experimentation, past 30 day use, and future use intent across different e-cigarette device types (e.g., disposables, pod/cartridge-based, tanks). Such research can inform targeted AYA e-cigarette messaging and curriculum used to prevent use among those who are susceptible or intending to use, to prevent continued use among those who have tried e-cigarettes, and to help stop use among those AYAs using in the past 30 days. In this study, we assess which sociodemographic factors are associated with e-cigarette (1) ever-use versus never-use, (2) susceptibility among never-users, (3) past 30-day use versus former use (ever-users who did not use in the past 30 days), and (4) intent to use e-cigarettes in the next six months among both former users and past 30-day users, by device type.

## 2. Methods

### 2.1. Participants and Recruitment

To recruit participants and conduct a national online survey from 6 May to 14 May 2020, we used Qualtrics recruitment and survey management technology. Qualtrics maintains a database of panel members who are recruited via gaming sites, social media, customer loyalty portals, and website intercept recruitment. Based on our non-random sampling design, Qualtrics provided its panel members with a link to a description of the survey. Informed consent was obtained from all participants involved in the study. Sampling quotas were used to recruit by age (i.e., an equal proportion of adolescents ages 13–17 years, emerging adults ages 18–20 years, and young adults ages 21–24 years); e-cigarette use (i.e., an equal proportion of ever-e-cigarette users to never-e-cigarette users); and sex and race/ethnicity (i.e., to balance percentage of participants per U.S. Census data). Additional information describing our convenience sample is available elsewhere [5,12,22]. Qualtrics monitored and controlled the quality of survey responses, since its services meet ESOMAR standards for social and behavioral research. We excluded participants who completed the self-administered survey in less than one-third of the average completion time. The study protocol was approved by the institutional review board at Stanford University.

### 2.2. Measures

#### 2.2.1. Sociodemographic Information

We collected sociodemographic information as follows. Age was assessed by asking participants: “How old are you today?” where they could select from 13–24 years. We then grouped sample participants by below 21 years (13–20 years old) and 21 and above (i.e., 21–24 years old), which is the minimum age of tobacco sales per federal law [23]. For sex, participants were asked, “How do you identify as?” with answer-choices as (1) Female, (2) Male, (3) Non-Binary/Other, (4) Choose to not specify/indicate and for sexual orientation, participants were asked, “Do you consider yourself to be: (1) Heterosexual or straight, (2) LGBTQ+, (3) Other, please specify,” with a text entry option. For race/ethnicity, participants were asked, “What is your race?” with answer choices as (1) American Indian or Alaska Native, (2) Asian, (3) African American or Black (AA/Black), (4) Native Hawaiian or Other Pacific Islander, (5) White, (6) More than one race, and (7) Prefer not to answer; and “What is your ethnicity?” with responses as (1) Hispanic or Latino or (2) Not Hispanic or Latino. We collapsed participant responses to (1) AA/Black non-Hispanic; (2) Asian/ Native Hawaiian or Pacific Islander, non-Hispanic; (3) Hispanic, non-AA/black; (4) Other/multiracial, non-Hispanic; and (5) White, non-Hispanic. Finally, for educational attainment we asked participants “What is the highest level of education obtained by your mother?” with answer choices as (1) don’t know, (2) completed high school or below, (3) started college, (4) completed college (2- or 4-year degree), and (5) graduate or professional degree (Masters, Ph.D., M.D., J.D., etc.).

#### 2.2.2. E-Cigarette Ever-Use and Past-30-Day Use of Different Device Types

We included a detailed, image-based description and examples of brands corresponding to each device type for disposable, pod/cartridge-based and other e-cigarette devices [24]. To collect data about different device types, participants were asked, “Have you EVER USED any of these products in your entire life?” for each of the following products: (1) disposable pod/cartridge-based vape, like Puff Bar or FOGG, even one or two puffs, (2) pod/cartridge-based vape, like JUUL or Suorin, even one or two puffs, (3) any other vape like mods, even one or two puffs?, with answer choices as “Yes” or “No.” If participants answered “Yes” to having ever used any of these devices, they were asked “During the LAST 30 DAYS, ON ABOUT HOW MANY DAYS did you use (for each device type selected)?” Participants were asked to select the number of days used from 0 to 30 days and enter “0” if they did not use the product in the last 30 days. In this study, “former users” were defined as ever-users of e-cigarettes who did not use e-cigarettes in the past 30 days.

#### 2.2.3. Intent to Use E-Cigarettes in the Future among Ever-Users

Depending on their previous device ever-used (see measures related to e-cigarette ever-use and past-30-day use of different device types above), participants were asked, “How likely is it that over the next six months you will use… (1) disposable vapes, like Puff Bar; (2) pod/cartridge-based vape, like JUUL or Suorin; (3) other vapes (like mods and non-disposable or pod/cartridge-based vapes) again?” Participants were asked to choose from: (a) very unlikely, (b) somewhat unlikely, (c) somewhat likely, or (d) very likely. We identified participants intending to use e-cigarettes in the future as those who indicated any response apart from “(a) very unlikely,” which represents a firm resolve not to use in the future.

#### 2.2.4. Susceptibility to Use E-Cigarette among Never-Users

If participants indicated that they had never used an e-cigarette device (disposable, pod/cartridge-based, or other), we asked the following concerning each device type: (1) “Do you think you will try it in the next month?” (2) “At any time in the next year do you think you will use it?” and (3) “If a friend offered it to you, would you use it?”with the following answer-choices provided: (a) definitely not, (b) probably not, (c) probably yes, (d) definitely yes. Then, participants were asked, (4) “Have you ever been curious about using it?”with answer-choices including (a) not at all curious, (b) a little curious, (c) somewhat curious, (d) very curious. We identified participants as susceptible to e-cigarette use if they expressed anything except a firm resolve against trying an e-cigarette (i.e., any answer apart from “(a) definitely not” as a response to questions 1–3 above) or were curious about e-cigarettes (i.e., any answer apart from “(a) not at all curious” as a response to question 4 above). These questions and coding were adapted from the Enhanced Susceptibility Index and other studies assessing e-cigarette susceptibility among never-users [18,25,26].

### 2.3. Analytic Strategy

Descriptive statistics assessed susceptibility to using in the future among never-users and intent to use in the future among former users (ever users who did not use in the past 30 days) and past-30-day users. We reported weighted percentages for comparisons between ever- and never-users given that we sampled for an equal proportion of ever-users to never-users. Unweighted ordered logistic regression models examined associations between sociodemographic factors and (1) susceptibility to using e-cigarettes among never users, (2) past-30-day versus former use among ever-users, (3) odds of using in the next six months among former users, and (4) odds of using in the next six months among past-30-day users for different device types, namely (a) disposables, (b) pod/cartridge-based and (c) other devices. Missing data were list-wise deleted, resulting in analytic samples as indicated. Weighted ordered logistic regression models examined the associations between sociodemographic factors and ever versus never use of e-cigarettes. These weights were designed to re-balance the deliberate oversampling of equal proportions of e-cigarette ever-users to never-users in our total sample. Thus, weights were applied to analyses concerning ever versus never e-cigarette users, and not in sub-analyses related to ever users (former users or past 30-day users). A detailed description of the weighting strategy is available elsewhere [12]. All regression models where we report adjusted odds ratios include age, sex, identifying as LGBTQ+, race/ethnicity and mother’s education. Data were analyzed using two-tailed statistical tests using Stata 15.1, and *p* values less than *p* < 0.05 were considered to be statistically significant.

## 3. Results

Our sample was comprised of 2168 never-users and 2183 ever-users of e-cigarettes (total n = 4351). In our total sample, 66.4% were 13–20 years old and 33.6% were 21–24 years old, 50.4% self-identified as female and 17.9% as LGBTQ+. In terms of race/ethnicity, 13.8% self-identified as AA/Black non-Hispanic, 15.2% as Hispanic or Latino, 6.1% as other/multi-race non-Hispanic, 4.8% as Asian/Pacific Islander non-Hispanic and the remaining 60.0% as White non-Hispanic. A detailed description of the total sample is available elsewhere [12]. Among ever-users, 906 self-reported having ever-used e-cigarettes but not in the past 30-days (former users) and 1086 self-reported having used e-cigarettes in the past 30 days.

As shown in Table 1, more than two-thirds of all former- and past-30-day-users in our sample were under 21 years of age, 71% of former users and slightly more than half of past-30-day users were females, and in descending order, White non-Hispanic, Hispanic non-AA/Black and AA/Black non-Hispanic who were both former users and past-30-day users were likely to use in the future. Among 1281 ever users of disposable devices, 45.4% were former users and 54.6% were past-30-day users. Among the 1694 ever users of pod/cartridge-based devices, 54.5% were former users and 45.5% were past 30-day users. Among the 1227 ever users of other types of devices, 59.6% were former users and 40.4% were past-30-day users. Among ever-users, 66.3% of former users and 94.5% of 1034 past-30-day users intended to use in the next six months.

Overall, 32.5% of 2031 never-users who answered questions related to susceptibility were susceptible to using any type of e-cigarette in the future. Regarding never-users’ specific susceptibility to different devices, 26.1% of disposable never-users were susceptible to using disposables, 30.4% of pod/cartridge-based never-users were susceptible to using pod/cartridge-based devices and 25.3% of other e-cigarette (like mods) never-users were susceptible to using other e-cigarette devices.

### Sociodemographic Factors Associated with Using Different Device Types

As shown in Table 2a, AYAs identifying as LGBTQ+ compared to non-LGBTQ+ were more likely to ever-use a pod/cartridge-based device (adjusted Odds Ratio (aOR) = 2.38, 1.07–5.30) and AYAs aged 13–20 versus 21–24 years-old were less likely to use other e-cigarette devices, like mods (aOR = 0.60, 0.43–0.85). Black/African American non-Hispanic AYAs were less likely to use pod/cartridge-based (aOR = 0.36, 0.16–0.80) and other e-cigarette devices (like mods) (aOR = 0.42, 0.20–0.84) compared to White non-Hispanic AYAs.

Table 2b shows that AYA never-users identifying as LGBTQ+ compared to non-LGBTQ+ showed a higher likelihood of susceptibility to using all e-cigarette device types (disposable (aOR = 1.50, 1.13–1.99); pod/cartridge-based (aOR = 1.51, 1.15–1.99); other e-cigarette devices (aOR = 1.61, 1.21–2.14)). Further, susceptibility to use all device types was more likely among AA/Black non-Hispanic AYAs compared to White non-Hispanic AYAs (disposable (aOR = 1.48, 1.09–2.00); pod/cartridge-based (aOR = 1.35, 1.00–1.81); other e-cigarette devices (aOR = 1.55, 1.14–2.09)). Hispanic non-AA/Black AYAs were more likely to be susceptible to using pod/cartridge-based devices (aOR = 1.38, 1.00–1.90) and other/multi-race non-Hispanic AYAs were more likely to be susceptible to using other e-cigarette devices (aOR = 1.98, 1.19–3.29) compared to White non-Hispanic AYAs. Having a mother who completed a college education compared to a high school level education made an individual less likely to use a disposable (aOR = 0.71, 0.54–0.94) or other e-cigarette device (aOR = 0.70, 0.53–0.93).

As shown in Table 2c, AYAs were more likely to be past 30-day users of disposables compared to former users if they identified as non-Hispanic multi-race/other AYAs compared to non-Hispanic White AYAs (disposable (aOR = 1.60, 1.04–2.45); pod/cartridge-based (aOR = 1.58, 1.08–2.30); other e-cigarette devices (aOR = 1.69, 1.09–2.62)). AYAs identifying as AA/Black non-Hispanic compared to White non-Hispanic AYAs were more likely to have used pod/cartridge-based devices in the past 30 days compared to former users (aOR = 1.51, 1.10–2.06) and other e-cigarette devices in the past 30 days compared to former users (aOR = 2.58, 1.76–3.79). For other e-cigarette devices, AYAs identifying as other/multi-race non-Hispanic compared to White non-Hispanic were more likely to have used other e-cigarettes devices in the past 30 days compared to former users (aOR = 1.89, 1.36–2.64). AYAs were less likely to be past-30-day users compared to former users if they were under 21 year of age versus 21 and above (disposable (aOR = 0.73, 0.56–0.94); pod/cartridge-based (aOR = 0.58, 0.46–0.73); other e-cigarette devices (aOR = 0.50, 0.38–0.64)) and identified as female compared to male (disposable (aOR = 0.61, 0.47–0.79); pod/cartridge-based (aOR = 0.50, 0.40–0.63); other e-cigarette devices (aOR = 0.43, 0.33–0.57)).

Table 3a shows that limited sociodemographic factors were associated with former users’ intent to use the same device in the future. AYA ever users of other e-cigarette devices were more likely to intend to use the same types of devices in the next six months if they were under 21 years compared to 21 and above (aOR = 1.53, 1.07–2.17). Among past-30-day users (see Table 3b), AYAs were less likely to use disposables in the next six months if they identified as AA/Black non-Hispanic (aOR = 0.34, 0.18–0.64) and less likely to use other e-cigarettes devices if they identified as Hispanic non-AA/Black (aOR = 0.45, 0.23–0.86) compared to White non-Hispanic AYAs.

## 4. Discussion

Our study provides data about sociodemographic factors associated with susceptibility, use and future intent to use different e-cigarette device types. Our cross-sectional survey study was conducted at a time when youth were reporting a rise in use of disposable devices, although pod/cartridge-based devices were still the most widely used device type overall [3]. Remarkably, sociodemographic factors were not associated with AYA ever-use of disposable devices; however, identifying as LGBTQ+ compared to other AYAs was associated with a higher likelihood of using pod/cartridge-based devices. Susceptibility among never-users appears more likely among youth identifying as LGBTQ+ for using disposable, pod/cartridge-based and other devices; however, identifying as LGBTQ+ was not a significant factor related to ever-users continuing to use in the past 30 days. Although identifying as AA/Black non-Hispanic, compared to White non-Hispanic, was associated with a lower likelihood of ever use of pod/cartridge-based and other e-cigarette devices, AA/Black non-Hispanic AYAs were more likely susceptible to using among never-users of all devices and past-30-day use versus former use for pod/cartridge-based and other e-cigarette devices among the AYAs in our study. Other/multiracial, non-Hispanic AYAs relative to White non-Hispanic AYAs were more likely to have used all device types in the past 30 days compared to former users and have greater susceptibility among never-users of other e-cigarette devices. Never-users were less likely to be susceptible to using disposables and other e-cigarette devices if their mothers had completed college-level education (indicative of higher socio-economic position) and disposables if they did not know their mother’s educational attainment. Underage youth who had ever experimented with other e-cigarette devices, like mods, but not in the past 30 days, were more likely to intend to use in the future compared to AYAs 21 years and above. Generally, past-30-day users of all device types were likely to intend to use their device type in the future regardless of sociodemographic factors.

Our data showing that AYAs who identify as LGBTQ+ have a higher likelihood of susceptibility to use different devices are consistent with previous studies [18,20,21]. The finding that AYAs identifying as LGBTQ+ were more likely to have ever used pod/cartridge-based devices is supported by evidence from a California-based study where participants identifying as LGBTQ+ were more likely to have used JUUL in the past 12 months [18]. Previous studies show that LGBTQ+ youth are more receptive to marketing compared to other youth [20]; thus, educational and prevention programs may be developed and tested for their effects on preventing and reducing e-cigarette susceptibility and use among LGBTQ+ youth. A recent study suggests that sexual minority young adults were more likely to be exposed to advertising in retail, gas station, or convenience stores [21], which may warrant educational content focused on dissecting marketing exposure and messages that are common in these locations.

Data from this study may be used to inform additional research and the development of targeted prevention of AYA e-cigarette use among susceptible sociodemographic groups. Our study shows that some racial/ethnic groups of AYAs are more likely to become past-30-day users, compared to those who had ever used but not in the past 30 days. Previous studies provide mixed evidence on whether AA/Black and other/multi-racial youth are more or less likely to use e-cigarettes compared to White non-Hispanic youth, with some variation by flavor categories [19,27,28]. Further, studies increasingly show that stress is a reason why AYAs report using e-cigarettes [18,29], and stress may disproportionately affect some racial and ethnic minorities and LGBTQ+ youth compared to other AYAs. Thus, there is a need for additional qualitative and quantitative research and prevention-focused studies to uncover why and how race/ethnicity plays a role in use of different device types. Our study also found no significant difference between those aged below 21 years and those 21 years and above in ever use and susceptibility to use pod/cartridge-based and disposable device types. Further, since young adults were more likely to use e-cigarettes in the past 30 days compared to those under 21 years, there is a need to focus our attention on preventing continuing use of e-cigarettes through age-appropriate prevention programs for young adults in addition to adolescents.

Our findings also highlight two key challenges in developing targeted prevention. First, the lack of a strong association between sociodemographic factors and use, susceptibility and future use intent of disposables emphasizes that all AYAs should be reached to prevent the use of disposables. Given that disposables are currently the most widely used products in the past 30 days among AYAs [4], we hypothesize that other, well-documented reasons and motivations, such as availability of flavors, concealability, susceptibility to marketing and peer pressure, and the FDA’s prioritized enforcement against pod/cartridge-based e-cigarette devices, are some reasons behind why AYAs use these devices [5]. Furthermore, we have limited literature on the extent to which AYAs’ risky behaviors, school performance, and parental monitoring [30] differentially impact their use of particular e-cigarette devices, whether exclusively or in combination with other devices. Second, limited insights are available in terms of sociodemographic factors that make AYA former and past-30-day e-cigarette users more likely to intend to use e-cigarettes in the future, suggesting a need for additional qualitative research on factors underlying repeated and/or continued use.

## 5. Limitations

This study relies on self-reported information from a convenience sample on sociodemographic factors, susceptibility, and use of different e-cigarette device types. These cross-sectional data cannot be used to establish causation between factors and e-cigarette use. Mother’s education was used as a proxy measure of socioeconomic position; in the future a more specific question on household income/prosperity may be used in line with the current literature. We believe that participants were adequately clear about the distinctions between different device types as we included a detailed, image-based description and examples of brands prior to questions about use. We did not include an analysis of sociodemographic factors associated with use of specific brands, as some brands manufacture multiple device types and research from this dataset indicates that most disposable users in this sample used Puff Bar and the majority of pod/cartridge-based users in this sample used JUUL [5]. We did not ask about brands used for the other e-cigarette device categories, including mods.

## 6. Conclusions

This study reports sociodemographic factors related to initiation, experimentation, past-30-day use and intended future use of different e-cigarette devices. Notably, this study shows that sociodemographic factors appear to have limited associations with ever-use of disposable e-cigarettes and generally on users’ intent to use e-cigarettes in the future. Given that disposables are currently the most popular type of e-cigarette device used by AYAs, a lack of association with sociodemographic factors highlights challenges in creating targeted prevention efforts for disposable devices. For other device types, our study found that LGBTQ+ youth compared to other AYAs were more likely to ever use pod/cartridge-based devices. Among never-users, LGBTQ+ youth were more likely to be susceptible to disposable, pod/cartridge-based and other e-cigarette devices. Other/multiracial, non-Hispanic AYAs compared to White non-Hispanic AYAs had a higher likelihood of continuing to use in the past 30 days compared to former use of all device types and being susceptible to using other e-cigarette devices like mods. AA/Black non-Hispanic AYAs compared to White non-Hispanic AYAs were more likely to be susceptible to using all types of e-cigarette devices as well as for continuing use in to the past 30 days for pod/cartridge-based and other devices, like mods and tanks. Youth aged under 21 who had used other e-cigarette devices like mods but not in the past 30 days were more likely to intend to use these devices in the future compared to youth 21 years and above. Generally, flavors, widespread marketing, peer use, concealability, and stress-relief are some reasons why AYAs use e-cigarettes. Our findings underscore that the different sociodemographic profiles of AYAs are related to the susceptibility and use of different device types, suggesting that users may have been targeted with these appealing characteristics. A nuanced understanding of which sociodemographic groups are likely to continue using products may help to focus prevention and cessation efforts on those who need them the most.

## Figures and Tables

**Table 1:** Participant characteristics by susceptibility among never-users and intended future use among ever-users and past-30-day users (n, unweighted %).

	Never-Users * (n = 2031)		Ever-Users, No Past 30-Day Use * (n = 606)		Past 30-Day Users * (n = 1034)	
	Not Susceptible (n = 1371)	Susceptible (n = 660)	Not Likely to Use in the Next 6 Months (n = 204)	Likely to Use in the Next 6 Months (n = 402)	Not Likely to Use in the Next 6 Months (n = 57)	Likely to Use in the Next 6 Months (n = 977)
Age						
13–20	893 (65.1)	461 (69.8)	164 (80.4)	325 (80.8)	42 (73.7)	697 (71.3)
21–24	465 (33.9)	195 (29.6)	40 (19.6)	73 (18.2)	15 (26.3)	274 (28.1)
Missing	13 (0.9)	4 (0.6)	0 (0.0)	4 (1.0)	0 (0.0)	6 (0.6)
Sex						
Male	421 (31.2)	208 (31.7)	56 (27.4)	104 (25.6)	25 (43.9)	382 (39.1)
Female	912 (66.9)	429 (65.4)	145 (71.1)	288 (71.9)	31 (54.4)	572 (58.5)
Other	25 (1.8)	19 (2.9)	3 (1.5)	10 (2.5)	1 (1.7)	22 (2.2)
Missing	0 (0.0)	0 (0.0)	0 (0.0)	0 (0.0)	0 (0.0)	1 (0.1)
LGBTQ						
Yes	192 (14.1)	122 (18.5)	36 (17.6)	98 (24.4)	13 (22.8)	201 (20.6)
No	1163 (85.7)	534 (81.5)	168 (82.3)	304 (75.6)	44 (77.2)	775 (79.3)
Missing	3 (0.2)	0 (0.0)	0 (0.0)	0 (0.0)	0 (0.0)	1 (0.1)
Race/ethnicity						
AA/Black, non-Hispanic	167 (12.3)	86 (13.1)	34 (16.6)	43 (10.8)	17 (29.8)	131 (13.5)
Asian/Native Hawaiian or Pacific Islander, non-Hispanic	34 (2.5)	23 (3.5)	19 (9.3)	24 (6.0)	3 (5.3)	71 (7.3)
Hispanic, non-AA/black	138 (10.2)	84 (12.8)	33 (16.2)	73 (18.3)	13 (22.8)	190 (19.6)
Other/multiracial, non-Hispanic	41 (3.0)	27 (4.1)	14 (6.9)	27 (6.8)	6 (10.5)	98 (10.1)
White, non-Hispanic	978 (72.0)	436 (66.5)	104 (51.0)	231 (58.0)	18 (31.6)	481 (49.5)
Mother’s education						
Started college	188 (13.8)	84 (12.8)	25 (12.2)	59 (14.8)	8 (14.0)	152 (15.6)
Completed college (2- or 4-year degree)	485 (35.7)	210 (32.0)	76 (37.2)	133 (33.4)	17 (29.8)	301 (31.0)
Graduate or professional degree (e.g., Masters, Ph.D., M.D., J.D., etc.)	256 (18.9)	139 (21.2)	36 (17.6)	81 (20.3)	16 (28.1)	219 (22.6)
High school or below	287 (21.1)	165 (25.1)	49 (24.0)	84 (21.1)	12 (21.0)	227 (23.4)
Don’t know	135 (9.9)	56 (8.5)	18 (8.8)	41 (10.3)	3 (5.3)	67 (6.9)
Missing	7 (0.5)	2 (0.3)	0 (0.0)	0 (0.0)	1 (1.7)	5 (0.5)

* Our sample comprised of 2168 total never users, of whom 2031 answered questions on susceptibility to use e-cigarettes. Similarly, our sample comprised of 2183 ever-users, including 906 ever e-cigarette users who did not use in the past 30 days and 1086 past-30-day e-cigarette users; questions about intended future use were answered by 606 ever e-cigarette users who did not use in the past 30 days and 1034 past-30-day e-cigarette users, respectively. Ns in the top row indicate those who answered the question on susceptibility and were likely to use in the next six months.

**Table 2:** Association between sociodemographic factors and… [Adjusted Odds Ratio (95%CI)] ^.

**(a)** **Ever-Users vs. Never Users of…**			
	**Disposables**	**Pod/Cartridge-Based**	**Other**
Age			
13–20	0.95 (0.67, 1.35)	1.10 (0.71, 1.68)	**0.60 (0.43, 0.85)**
21–24	Ref	Ref	Ref
Sex			
Female	0.82 (0.49, 1.35)	1.27 (0.70, 2.29)	0.80 (0.49, 1.30)
Other	1.04 (0.44, 2.47)	0.87 (0.32, 2.38)	1.38 (0.60, 3.20)
Male	Ref	Ref	Ref
LGBTQ			
Yes	1.18 (0.60, 2.32)	**2.38 (1.07, 5.30)**	1.40 (0.76, 2.58)
No	Ref	Ref	Ref
Race/ethnicity			
AA/Black, non-Hispanic	0.54 (0.25, 1.15)	**0.36 (0.16, 0.80)**	**0.42 (0.20, 0.84)**
Asian/Native Hawaiian or Pacific Islander, non-Hispanic	0.93 (0.57, 1.54)	1.32 (0.69, 2.54)	0.79 (0.48, 1.28)
Hispanic, non-AA/black	0.80 (0.41, 1.55)	0.93 (0.41, 2.07)	1.25 (0.65, 2.39)
Other/multiracial, non-Hispanic	0.40 (0.15, 1.03)	1.19 (0.37, 3.88)	0.80 (0.27, 2.34)
White, non-Hispanic	Ref	Ref	Ref
Mother’s education			
Started college	1.33 (0.58, 3.07)	1.26 (0.46, 3.41)	0.46 (0.20, 1.06)
Completed college (2- or 4-year degree)	0.94 (0.48, 1.81)	1.22 (0.51, 2.87)	0.63 (0.33, 1.19)
Graduate or professional degree (Masters, Ph.D., M.D., J.D., etc.)	0.75 (0.33, 1.67)	0.57 (0.22, 1.48)	0.57 (0.26, 1.22)
Don’t know	0.66 (0.27, 1.59)	0.45 (0.16, 1.20)	0.77 (0.32, 1.83)
High school or below	Ref	Ref	Ref
**(b)** Susceptibility to using different e-cigarette devices among never users *…			
	**Disposables (n = 2003)**	**Pod/Cartridge-Based** **(n = 1917)**	**Other (n = 1998)**
Age			
13–20	1.08 (0.87, 1.35)	1.23 (0.99, 1.53)	0.93 (0.74, 1.17)
21–24	Ref	Ref	Ref
Sex			
Female	0.98 (0.78, 1.22)	0.93 (0.75, 1.16)	0.83 (0.67, 1.04)
Other	0.77 (0.37, 1.60)	1.03 (0.52, 2.02)	1.03 (0.53, 2.01)
Male	Ref	Ref	Ref
LGBTQ			
Yes	**1.50 (1.13, 1.99)**	**1.51 (1.15, 1.99)**	**1.61 (1.21, 2.14)**
No	Ref	Ref	Ref
Race/ethnicity			
AA/Black, non-Hispanic	**1.48 (1.09, 2.00)**	**1.35 (1.00, 1.81)**	**1.55 (1.14, 2.09)**
Asian/Native Hawaiian or Pacific Islander, non-Hispanic	1.80 (1.02, 3.17)	1.35 (0.76, 2.41)	1.39 (0.76, 2.54)
Hispanic, non-AA/black	1.37 (0.99, 1.91)	**1.38 (1.00, 1.90)**	1.33 (0.95, 1.86)
Other/multiracial, non-Hispanic	1.54 (0.90, 2.62)	1.13 (0.67, 1.92)	**1.98 (1.19, 3.29)**
White, non-Hispanic	Ref	Ref	Ref
Mother’s education			
Started college	0.92 (0.65, 1.28)	0.87 (0.61, 1.22)	0.79 (0.55, 1.12)
Completed college (2- or 4-year degree)	**0.71 (0.54, 0.94)**	0.89 (0.68, 1.16)	**0.70 (0.53, 0.93)**
Graduate or professional degree (Masters, Ph.D., M.D., J.D., etc.)	0.93 (0.68, 1.26)	1.04 (0.77, 1.42)	0.99 (0.73, 1.34)
Don’t know	**0.52 (0.34, 0.78)**	0.70 (0.48, 1.04)	0.69 (0.46, 1.03)
High school or below	Ref	Ref	Ref
**(c)** Past 30-day users vs. former users (ever users who did not use in the past 30 days) among ever-users * of…			
	**Disposables (n = 1266)**	**Pod/Cartridge-Based** **(n = 1679)**	**Other (n = 1210)**
Age			
13–20	**0.73 (0.56, 0.94)**	**0.58 (0.46, 0.73)**	**0.50 (0.38, 0.64)**
21–24	Ref	Ref	Ref
Sex			
Female	**0.61 (0.47, 0.79)**	**0.50 (0.40, 0.63)**	**0.43 (0.33, 0.57)**
Other	0.53 (0.24, 1.18)	0.65 (0.32, 1.33)	0.45 (0.19, 1.05)
Male	Ref	Ref	Ref
LGBTQ			
Yes	1.04 (0.78, 1.40)	1.01 (0.78, 1.30)	1.24 (0.91, 1.70)
No	Ref	Ref	Ref
Race/ethnicity			
AA/Black, non-Hispanic	1.23 (0.86, 1.76)	**1.51 (1.10, 2.06)**	**2.58 (1.76, 3.79)**
Asian/Native Hawaiian or Pacific Islander, non-Hispanic	0.87 (0.55, 1.37)	1.18 (0.79, 1.78)	1.13 (0.67, 1.91)
Hispanic, non-AA/black	1.31 (0.95, 1.80)	1.21 (0.92, 1.59)	**1.89 (1.36, 2.64)**
Other/multiracial, non-Hispanic	**1.60 (1.04, 2.45)**	**1.58 (1.08, 2.30)**	**1.69 (1.09, 2.62)**
White, non-Hispanic	Ref	Ref	Ref
Mother’s education			
Started college	1.36 (0.93, 1.98)	1.06 (0.76, 1.48)	0.81 (0.54, 1.21)
Completed college (2- or 4-year degree)	0.85 (0.62, 1.16)	0.85 (0.64, 1.12)	0.83 (0.59, 1.15)
Graduate or professional degree (Masters, Ph.D., M.D., J.D., etc.)	1.27 (0.90, 1.81)	1.00 (0.74, 1.35)	1.18 (0.82, 1.70)
Don’t know	0.81 (0.50, 1.29)	0.73 (0.49, 1.09)	0.71 (0.44, 1.14)
High school or below	Ref	Ref	Ref

^ Bold indicates significance at *p* < 0.05; * Unweighted.

**Table 3:** Odds of using in the next 6 months among … [unweighted AOR (95%CI)] ^.

**(a)** **Former Users**			
	**Disposables** **(n = 572)**	**Pod/Cartridge-Based** **(n = 897)**	**Other** **(n = 701)**
Age			
13–20	1.14 (0.78, 1.67)	1.17 (0.84, 1.62)	**1.53 (1.07, 2.17)**
21–24	Ref	Ref	Ref
Sex			
Female	1.05 (0.72, 1.55)	0.78 (0.57, 1.08)	1.01 (0.71, 1.44)
Other	1.79 (0.52, 6.15)	1.49 (0.52, 4.28)	2.26 (0.75, 6.83)
Male	Ref	Ref	Ref
LGBTQ			
Yes	1.42 (0.92, 2.20)	1.36 (0.97, 1.89)	1.37 (0.94, 2.00)
No	Ref	Ref	Ref
Race/ethnicity			
AA/Black, non-Hispanic	0.60 (0.35, 1.04)	0.96 (0.61, 1.49)	0.99 (0.57, 1.71)
Asian/Native Hawaiian or Pacific Islander, non-Hispanic	0.99 (0.53, 1.87)	0.71 (0.40, 1.27)	1.73 (0.91, 3.28)
Hispanic, non-AA/black	0.95 (0.59, 1.55)	1.04 (0.71, 1.50)	1.05 (0.67, 1.64)
Other/multiracial, non-Hispanic	0.77 (0.38, 1.54)	1.17 (0.67, 2.05)	1.20 (0.66, 2.18)
White, non-Hispanic	Ref	Ref	Ref
Mother’s education			
Started college	1.22 (0.68, 2.18)	1.50 (0.94, 2.38)	1.22 (0.74, 2.00)
Completed college (2- or 4-year degree)	0.96 (0.61, 1.50)	1.00 (0.69, 1.46)	1.12 (0.74, 1.69)
Graduate or professional degree (Masters, Ph.D., M.D., J.D., etc.)	1.37 (0.80, 2.33)	1.30 (0.86, 1.96)	1.38 (0.86, 2.21)
Don’t know	0.91 (0.47, 1.79)	0.98 (0.57, 1.65)	1.02 (0.57, 1.84)
High school or below	Ref	Ref	Ref
**(b)** **Past 30-day users of…**			
	**Disposables** **(n = 670)**	**Pod/Cartridge-Based** **(n = 736)**	**Other** **(n = 464)**
Age			
13–20	0.96 (0.58, 1.60)	1.04 (0.67, 1.61)	0.58 (0.33, 1.01)
21–24	Ref	Ref	Ref
Sex			
Female	1.36 (0.83, 2.21)	1.21 (0.79, 1.85)	0.94 (0.54, 1.63)
Other	1.27 (0.25, 6.50)	1.67 (0.35, 7.99)	0.60 (0.10, 3.39)
Male	Ref	Ref	Ref
LGBTQ			
Yes	0.75 (0.42, 1.33)	0.80 (0.48, 1.33)	0.81 (0.43, 1.55)
No	Ref	Ref	Ref
Race/ethnicity			
AA/Black, non-Hispanic	**0.34 (0.18, 0.64)**	0.63 (0.35, 1.13)	0.51 (0.24, 1.06)
Asian/Native Hawaiian or Pacific Islander, non-Hispanic	0.97 (0.31, 2.99)	1.46 (0.58, 3.64)	1.01 (0.28, 3.68)
Hispanic, non-AA/black	0.59 (0.31, 1.09)	0.73 (0.42, 1.25)	**0.45 (0.23, 0.86)**
Other/multiracial, non-Hispanic	0.56 (0.26, 1.20)	0.91 (0.43, 1.89)	0.71 (0.28, 1.81)
White, non-Hispanic	Ref	Ref	Ref
Mother’s education			
Started college	1.16 (0.56, 2.43)	1.69 (0.80, 3.57)	0.96 (0.39, 2.31)
Completed college (2- or 4-year degree)	1.41 (0.71, 2.80)	1.14 (0.64, 2.02)	0.88 (0.43, 1.82)
Graduate or professional degree (Masters, Ph.D., M.D., J.D., etc.)	0.78 (0.40, 1.49)	0.81 (0.45, 1.44)	0.84 (0.40, 1.76)
Don’t know	0.76 (0.30, 1.88)	0.41 (0.20, 0.84)	0.56 (0.21, 1.45)
High school or below	Ref	Ref	Ref

^ Bold indicates significance at *p* < 0.05.

## Data Availability

The data presented in this study are available on request from the corresponding author. The data are not publicly available due to their containing information that could compromise the privacy of research participants.
